# High-Molecular-Weight Glutenin Subunits: Genetics, Structures, and Relation to End Use Qualities

**DOI:** 10.3390/ijms22010184

**Published:** 2020-12-26

**Authors:** Yi Li, Jiahui Fu, Qun Shen, Dong Yang

**Affiliations:** 1 Beijing Key Laboratory of Functional Food from Plant Resources, College of Food Science & Nutritional Engineering, China Agricultural University, 17 East Tsinghua Rd., Beijing 100083, China; sy20193061047@cau.edu.cn (Y.L.); s20193060969@cau.edu.cn (J.F.); 2 Xinghua Industrial Research Centre for Food Science and Human Health, China Agricultural University, Xinghua 225700, China; 3 Key Laboratory of Plant Protein and Grain Processing, National Engineering Research Center for Fruit and Vegetable Processing, China Agricultural University, 17 East Tsinghua Rd., Beijing 100083, China; shenqun@cau.edu.cn

**Keywords:** HMW-GS, end use qualities, interchain disulfide bonds, secondary structures

## Abstract

High-molecular-weight glutenin subunits (HMW-GSs) are storage proteins present in the starchy endosperm cells of wheat grain. Encoding the synthesis of HMW-GS, the *Glu-1* loci located on the long arms of group 1 chromosomes of the hexaploid wheat (1A, 1B, and 1D) present multiple allelism. In hexaploid wheat cultivars, almost all of them express 3 to 5 HMW-GSs and the *1Ay* gene is always silent. Though HMW-GSs are the minor components in gluten, they are crucial for dough properties, and certain HMW-GSs make more positive contributions than others. The HMW-GS acts as a “chain extender” and provides a disulfide-bonded backbone in gluten network. Hydrogen bonds mediated by glutamine side chains are also crucial for stabilizing the gluten structure. In most cases, HMW-GSs with additional or less cysteines are related to the formation of relatively more or less interchain disulfide bonds and HMW-GSs also affect the gluten secondary structures, which in turn impact the end use qualities of dough.

## 1. Introduction

Wheat is one of the most widely grown cereal crops worldwide with hexaploid wheat (*Triticum aestivum* L., AABBDD) being a representative one. According to the Agricultural Market Information System (AMIS), the estimate of world wheat production is 764.9 million tons in 2020 to 2021 [1]. Take China as an example, as the world’s largest wheat producer, China produced 131 million tons of wheat and consumed 128 million tons in 2019, among which 67.7% was used for food and 15.0% was used for feed [2]. Protein makes up approximately 8% to 20% of the total mass of mature wheat grains [3]. Based on their solubility, protein in wheat grain can be categorized into four classes: albumin, globulin, gliadin, and glutenin [4]. The latter two, gliadin and glutenin, are the major components of gluten, which are similar in content and together accounts for approximately 80–85% of wheat protein [3]. The protein of wheat grain, especially gluten protein, is generally regarded as one of the most important factors determining dough properties and bread qualities [5,6,7].

Defined as the remaining rubbery mass after gently washing wheat dough to remove the bulk starch granules and water-soluble components, gluten usually contains approximately 75–85% protein on a dry weight basis, depending on the thoroughness of washing [8]. Glutenin subunits are the monomeric component of a gluten polymer in dough through covalent disulfide linkage and noncovalent interactions including hydrogen bonding and hydrophobic interaction, forming a tight gluten network backbone and providing dough elasticity as well as dough strength. On the other hand, most gliadins exist in their monomeric form, affording dough viscosity and extensibility via electrostatic interactions and hydrogen bonding [9,10]. Gliadins can be further separated from gluten by dissolving in 60% to 70% (*v*/*v*) ethanol solution. Besides, the solubility of glutenin subunits from gluten would be similar to gliadins in aqueous alcohols if the disulfide bonds are reduced [8]. Based on their electrophoresis mobility on a sodium dodecyl sulfate-polyacrylamide gel electrophoresis (SDS-PAGE), reduced glutenin from dough can be further categorized into High-molecular-weight glutenin subunits (HMW-GSs, MW of 67,000–90,000 Da) and low-molecular-weight glutenin subunits (LMW-GSs, MW of 30,000–45,000 Da) [11,12,13,14]. Though HMW-GSs are minor components in terms of mass (approximately 10% of gluten protein), they are highly related to end use quality [8]. This review aims to describe the genetics and different levels of structures of HMW-GS, and their relationships to dough and bread-making qualities.

## 2. Genetics of HMW-GS

**Figure 1:**
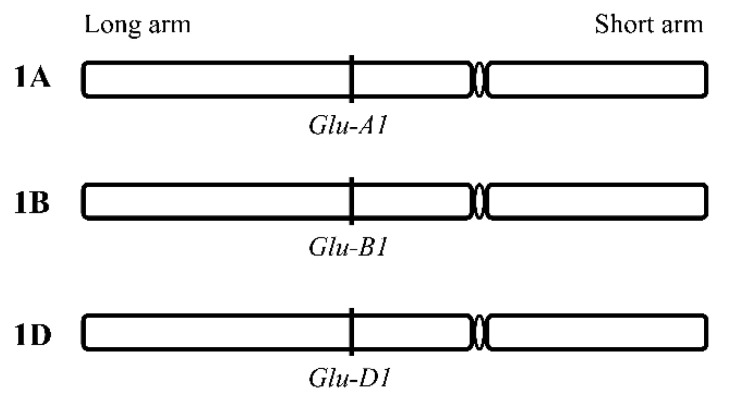
Schematic diagram of the gene loci of a high-molecular-weight glutenin subunit (HMW-GS) in wheat chromosome 1: the genes coding the synthesis of HMW-GS are located on the long arms of group 1 chromosomes 1A, 1B, and 1D.

**Figure 2:**
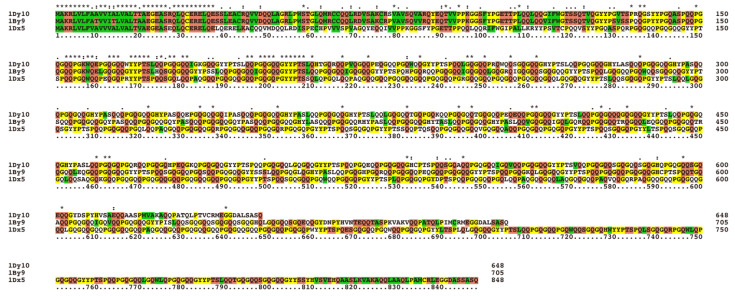
“*” indicates identical amino acid residues, “꞉” indicates similar amino acid residues, and “ꞏ” indicates less similar amino acid residues. Yellow indicates amino acid residues G, P, C, H, Y; red indicates amino acid residues T, S, N, Q, E, D, K, R; green indicates amino acid residues W, L, V, I, F, A, M, C.

It is now firmly established that genes named *Glu-1* loci encoding the synthesis of HMW-GS (*Glu-A1*, *Glu-B1*, and *Glu-D1*) are located on the long arms of group 1 chromosomes of the hexaploid wheat (1A, 1B, and 1D) (Figure 1) [15,16,17,18]. Each locus includes 2 genes linked together encoding 2 different types of HMW-GS, the x-type subunits with a relatively higher molecular weight and the y-type subunits which exhibit relatively higher electrophoretic mobility on the SDS-PAGE [12,13,14,19,20,21]. Moreover, subloci differences are greater than homoeoallelic differences. For example, differences between *Glu-D1x* genes (genes encoding x-type subunits) and *Glu-D1y* genes (genes encoding y-type subunits) are greater than differences between *Glu-D1y* genes and *Glu-B1y* genes, as shown in Figure 2 [22,23].

### 2.1. Allelic Variations of Glu-1 Loci

The *Glu-1* loci present multiple allelism: 3 alleles at the *Glu-1A* loci, 11 alleles at the *Glu-1B* loci, and 6 alleles at the *Glu-1D* loci were systematically reported by isolating HMW-GSs from SDS-PAGE in 1983, in which the numbering system developed to identify HMW-GS and provide a chromosomal location of the genes is currently still in use [24]. Polymerase chain reaction (PCR) and immunoassay using specific monoclonal antibodies are alternative ways of isolating novel genes encoding HMW-GS [25,26,27,28]. Since the publication of the catalog of gene symbols for wheat, more than 100 allelic variations at the *Glu-1* loci have been identified [29]. According to the collections of the HMW-GS alleles from over 7830 cultivars/lines, there is no doubt that the *Glu-1* loci exhibits high genetic polymorphisms [30]. In particular, the *Glu-1B* and *Glu-1D* alleles display higher variable frequency while that of the *Glu-1A* alleles is a much lower. Recently, a number of novel *Glu-1* genes from wheat landraces or related species have been identified to be of potential application values to wheat breeding, and efforts have also been made to transfer desirable *Glu-1* alleles to cultivated wheat by wide hybridization and chromosomal engineering approaches [14,31,32,33,34,35,36].

Working on HMW-GS allelic variations to improve wheat bread-making qualities has been an attractive research topic in the past several decades. Some allelic variations make greater contributions to dough elasticity and strength as well as loaf volume than others. In general, the *Glu-D1* loci exhibited the most significant effect on dough and bread-making properties, followed by *Glu-B1* and *Glu-A1* [37,38,39]. Furthermore, several studies found that both *Glu-D1* and *Glu-B1* exhibit significant effects on dough qualities on their own, whereas the effects of *Glu-A1* loci depend on the presence of other *Glu-1* subunits [40].

As for specific alleles encoding HMW-GSs, the *Glu-A1a* allele encoding 1Ax1 and the *Glu-D1d* allele encoding 1Dx5 and 1Dy10 were reported to be associated with better bread-making qualities, and the beneficial effects of these two alleles are additive [19]. Later, in 1987, after analysis of 84 hexaploid wheat varieties, the *Glu-1* quality scores assigned to each HMW-GS and corresponding alleles were reported. Among the alleles reported in the research, wheat containing the *Glu-D1d* allele encoding 1Dx5 and 1Dy10 represents the highest score, corresponding to higher bread-making qualities, while *Glu-A1c*, *Glu-B1a*, *Glu-B1d*, and *Glu-D1c* represent the lowest scores, corresponding to poor bread-making qualities [16]. By comparing the rapid mix test score from 153 German wheat varieties, it was discovered that the bread-making quality was positively influenced by alleles encoding subunits 1Ax1, 1Ax2*, 1Bx7 + 1By9, 1Bx14 + 1By15, 1Bx17 + 1By18, and 1Dx5 + 1Dy10 [41]. Meanwhile, by studying the deletion of combinations of HMW-GS loci, contributions of each HMW-GS to dough processing properties were ranked in the following order: 1Dx5 + 1Dy10 > 1Bx17 + 1By18 > 1Ax1 + Null [40]. Nevertheless, several studies argue that all HMW-GSs contribute positively to dough or bread-processing quality but only differ in magnitude, since the absence of HMW-GS with weaker effects, such as 1Dx2, 1Dy12, 1Bx20, and 1By20, also led to inferior flour-processing quality in wheat mutants [42,43,44,45,46].

### 2.2. Gene Expression

The mechanism that regulates HMW-GS expression remains largely unclear [47]. Some conserved *cis*-acting elements related to the expression of storage proteins have been identified in HMW-GS gene promoters [47,48,49,50]. To date, storage protein activator (SPA), a member of the basic leucine zipper (bZIP) family, has been identified to play a key role in the regulation of wheat grain storage protein synthesis [51,52,53,54,55]. From a theoretical point of view, the hexaploid wheat cultivars could express 6 different HMW-GSs, 1Ax, 1Ay, 1Bx, 1By, 1Dx, and 1Dy, while in fact, owing to gene silencing, almost all of them express 3 to 5 HMW-GSs depending on wheat cultivars except the few hexaploid wheat cultivars expressing all 6 HMW-GSs [56,57,58]. The expression frequencies of 1Dx, 1Dy, and 1Bx are usually the highest, while the 1Ax and 1By subunits sometimes are not expressed at all. On the other hand, the 1Ay subunit is often not expressed in the hexaploid wheat, whereas its expression is frequently reported in diploid and tetraploid wheat [19,59,60,61].

#### 2.2.1. Progress on the *Ay* Gene

Since *1Ay* genes show a high frequency of silence in the hexaploid wheat, researchers have focused on their promoters and coding region structures to explore probable causes. Comparing the 5′ upstream promoter region of the active gene encoding 1By9 with the same region from a silent y-type gene derived from chromosome 1A, it was discovered that there is a deletion of 85 bp bases in the latter, which was also found in the 5′ upstream promoter region of the inactive *Ay1d* gene from the cultivated emmer (*Triticum dicoccum*, 2*n* = 4x = 28, AABB) [62,63]. However, analysis of 141 accessions of diploid and tetraploid wheats found this 85 bp deletion to be present in the promoter regions of all 9 *1Ay* genes, regardless of whether these genes are active or not [64]. Researchers later constructed two promoter–GUS (β-glucuronidase) reporter gene chimeric vectors, pBI121-Ay-GUS and pBI121-Dx5-GUS, in which the native promoters were replaced by promoters from an inactive *1Ay* gene and an active gene encoding 1Dx5, respectively. The transient expression of promoter–GUS constructs indicated that the *1Ay* promoter can drive the expression of the GUS gene, verifying that 85 bp deletion in the promoter is not associated with inactivation of *1Ay* genes [60].

As for the coding region, an 8-kb insertion retroelement in a cloned pseudo *1Ay* gene from wheat cultivar Chinese Spring (*Triticum aestivum* L., AABBDD), termed wheat insertion sequence-2 (Wis-2), was reported, which interrupts the coding sequence [62]. Similarly, the *Ay* gene coding sequence in a tetraploid wheat cultivar, *T. turgidum* (AABB), is also disrupted by an insertion of a Wis retroelement, Wis-3, which may result in silencing of the *Ay* gene [65]. Besides the above, many scholars have focused on the stop codons in the coding region as well. Translations of three silenced *Ay* genes from the diploid and tetraploid wheat species, together with silencing of *1Ay* (from hexaploid wheat cv. Cheyenne) were disrupted by premature stop codons [64]. This is consistent with a previous study showing that the coding region of an inactive *Ay* gene, namely *Ay1^d^*, contains four stop codons [63]. In conclusion, these genes were highly unlikely to be expressed with full length and defects in the coding regions could be responsible for silencing of the *1Ay* genes.

Since the *Glu-1Ay* gene is usually silenced in hexaploid wheat, the utilization of active *1Ay* genes might be an effective strategy for improving flour quality. An active allele encoding 1Ay subunits was successfully integrated from wild emmer wheat (*Triticum turgidum* ssp. *dicoccoides*) into the hexaploid wheat (*Triticum aestivum*) [66]. In this study, the selected line TaAy7-40, which expresses 1Ay subunits stably, was reported to possess better processing quality. Two Swedish hexaploid wheat lines (W29323 and W3879) were found to contain both active *1Ax* and *1Ay* genes and the corresponding expressed subunits were initially named 21* and 21*y, which were later found inherited as a pair [57]. Moreover, the allele encoding 1Ax21* and 1Ay21* were also integrated into two Australian wheat cultivars and was found to have no significant alterations in their agronomic traits. However, the integration of the Ay subunit showed positive effects in protein and gluten content, protein composition, dough mixing properties, and Zeleny sedimentation values [67]. In 2019, scholars reported that expression of the gene encoding 1Ay21* has the potential to simultaneously increase protein content and grain yield under certain environment [68]. Thus, the active *Glu-1Ay* allele might be of potential value in breeding aiming to improve wheat flour quality.

#### 2.2.2. Progress on the *Bx7* Gene

Among various kinds of alleles, researchers discovered a unique *Glu-B1al* allele encoding overexpression of 1Bx7 (Bx7^OE^). This allele is highly associated with improved dough strength, which has been found in many cultivars and landraces [69,70,71,72,73]. However, an Australian cultivar H45, though confirmed to overexpress Bx7 subunits, had relatively low un-extractable polymeric gluten (an indicator of weak dough), the corresponding allele of which was then designated as *Glu-B1br* [74].

Compared to the gene encoding normal 1Bx7 subunits, the gene encoding 1Bx7^OE^ shows an 18 bp nucleotide duplication in the coding region and a 43 bp insertion in the matrix-attachment region (MAR) upstream to the gene promoter [75,76]. However, evidence indicated that these 18- and 43-bp sequence insertions are not associated with the high expression levels of 1Bx7 because the 18- and 43-bp indels were also found in accessions other than Bx7^OE^ [77]. There is also evidence that the *Glu-B1al* allele includes two copies of its x-type glutenin gene [71]. Later, by sequencing a bacterial artificial chromosome (BAC) clone encompassing the *Glu-B1* locus, scholars reported a 10.3-kb segmental duplication including the *Bx7* gene and a flanking long terminal repeat (LTR) retroelement [78]. Meanwhile, according to research collecting a large amount of diploid, tetraploid, and hexaploid accessions, the LTR retroelement/duplication genomic structure was not found in accessions without Bx7 overexpression. These results indicated that gene duplication at the *Glu-B1* locus mediated by insertion of a retroelement may lead to overexpression of the Bx7 subunits [77].

## 3. Structures of HMW-GS

### 3.1. The Primary Structures

The mature HMW-GSs consist of three structural domains: a nonrepetitive N-terminal domain comprising approximately 81–104 residues and a C-terminal domain of 42 residues, flanking a repetitive central domain with 481 to 872 residues [79]. The repetitive central domain, as the largest part of HMW-GS, is identified by three types of primary repeat units in the x-type subunits and two types of primary repeat units in the y-type subunits. In the x-type subunits, the repeat units are the tripeptides (GQQ), hexapeptides (PGQGQQ), and nonapeptides (GYYPTSPQQ). In the y-type subunits, the repeat units are the hexapeptides (PGQGQQ) and nonapeptides (GYYPTSLQQ) [80,81,82]. Note that the tripeptide and hexapeptides of the x-type subunit are always present in tandem with each other [83]. While the N- and C-terminal regions are quite conservative, variations in the central repetitive domain, particularly the number of tripeptides and hexapeptides, are the main causes of differences in subunit size [79,82,84,85]. Meanwhile, as indicated by the amino acid composition, the central repetitive domain exhibited hydrophilic properties while the N-terminal and C-terminal domain exhibited hydrophobic characteristics [81,82]. A recent study shows that the central repetitive domain could be folded in the action of wheat protein disulfide isomerase, which serves as the basis of dough extension [86].

### 3.2. Cysteine Residues and Disulfide Bonds

**Figure 3:**
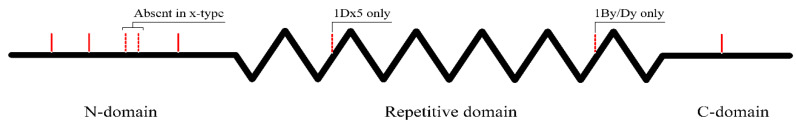
Schematic representation of the distribution of cysteine residues in x- and y-type HMW-GSs: the HMW-GS is comprised of three domains, the N- and C- terminal domains where most cysteine residues (red lines) are located, flanking a central repetitive domain. Two cysteine residues in the N-terminal of the y-type HMW-GSs are absent in the x-type. In HMW-GS 1Dx5, there is one additional cysteine residue in the repetitive domain near the N-terminal side, and there is an additional cysteine residue in the repetitive domain of the HWM-GSs 1By and 1Dy near the C-terminal side.

The distribution of cysteine residues in typical x-type and y-type subunits are shown in Figure 3. In general, most x-type subunits contain four cysteines, three in the N-terminal domain and one in the C-terminal domain [31,82]. The 1Dx5 subunit contains an additional cysteine at the beginning of the repetitive domain, while the 1Bx14 and 1Bx20 subunits contain only two cysteine residues, one in the N-terminal region and the other in the C-terminal domain [87,88,89,90,91]. A typical y-type subunit contains seven cysteines: five in N-terminal domains, one in the central repetitive domain, and one in the C-terminal domain [22,31]. Cysteine residues in the central domain of all y-type subunits usually do not exist in the x-type subunits.

Though cysteines are less abundant in HMW-GS, they are extremely crucial to the structure and functions of gluten [92]. Cysteines in the HMW-GSs form either intrachain disulfide bonds or interchain disulfide bonds with other HMW-GSs or LMW-GSs through oxidation, e.g., by catalysis of wheat protein disulfide isomerase [6,83,93,94]. In general, the HMW-GS acts as the “chain extender” of the gluten network owing to the fact that, in most cases, each HMW-GS can provide at least two cysteines participating in the formation of interchain disulfide bonds to form larger gluten aggregates, and it can be further improved by the addition of a so-called flour improver, which are oxidants in nature (e.g., azodicarbonamide and ascorbic acid) [8,95]. According to a structural model for wheat gluten, the HMW-GS provides a disulfide-bonded backbone which forms the basis for “branches” of LMW-GS linked by interchain disulfide bonds and interacts with gliadins by noncovalent interactions [96]. In addition, interchain disulfide bonds play an important role in stabilizing HMW-GS polymers [97]. For these contributing to gluten formation, the disulfides bonds are called “rheologically active” and others are “rheologically inactive”, and the former is catalyzed by wheat protein disulfide isomerase [98]. In FT-Raman Spectroscopy, the band appearing at 497 cm^−1^ is related to the interchain disulfide bond. Studies have shown that the content of interchain disulfide bond is positively associated with dough properties [99,100,101]. However, up to now, interchain bonds related to HMW-GS extracted from gluten have only been found: (1) between cysteines in a y-type HMW-GS N-terminal domain and the corresponding residue of another y-type HMW-GS, which are connected in parallel; (2) between the cysteine of a y-type HMW-GS repetitive central domain and a cysteine in LMW-GS; (3) and between the additional cysteine of 1Dx5 and the cysteine of an x-type HMW-GS C-terminal domain. Intrachain disulfide bonds related to HMW-GS extracted from gluten have only been between the adjacent cysteines of the N-terminal domain of 1Bx7, which is in line with a molecular model [102,103,104,105]. Other scholars have also proposed two possible patterns of disulfide bond formation according to the homology modeling and molecular dynamics simulations of the N-terminal domain of 1Dy10 [106]. Later in 2017, disulfide linkages in a recombinant N-terminal domain of 1Dx5 (1Dx5-N), which contains three cysteine residues (Cys10, Cys25, and Cys40), were dissected by site-directed mutagenesis and liquid chromatograph-mass spectrometer/mass spectrometry (LC-MS/MS). According to the LC-MS/MS results, intermolecular linkages were found in many combinations among Cys10, Cys25, and Cys40, indicating that the disulfide linkages between Cys10, Cys25, and Cys40 were not conserved strictly. Meanwhile, it was identified that Cys10 and Cys40 were the active sites for intermolecular linkages and that intramolecular linkages were only found between Cys25 and Cys40, which is contradictory to a molecular model of 1Dx5-N with no formation of intramolecular disulfide bonds by prediction [105,107]. Besides, the addition of 1Dx5-N greatly enhanced the formation of a gluten network through disulfide bonds cross-linking and hydrophobic interactions, which exerted a synergistic effect and thus contributed to the superior improvement in dough qualities [108].

It has been reported that, in most cases, HMW-GSs with additional or less cysteines were supposed to give rise to relatively higher or poorer qualities. The additional cysteine in 1Dx5, which might form another interchain bond, is supposed to be responsible for the superior bread properties exhibited in cultivars expressing 1Dx5 subunits [101,103,109]. As for HMW-GSs with less cysteine residues, scholars compared the effects of 1Bx20 subunit with two cysteine residues versus subunit 1Bx7 containing four cysteine residues and discovered that the wheat line carrying a 1Bx20 subunit exhibited less large glutenin polymer formation and poorer mixographic parameters [110]. Similar results have been obtained that incorporation of 1Bx20 subunits to base flour only resulted in small effects, either positive or negative, on dough strength and stability [25]. Meanwhile, 1Bx14*, which contains only two cysteine residues resulting in less formation of intermolecular disulfide bonds, is suggested to be partially the reason of poor milling quality [111]. These experiments fully demonstrated a role of the number of cysteine residues in determining the formation of glutenin polymers and subsequent rheology parameters of dough.

In addition to cysteine, glutathione (GSH), which is naturally present in wheat flour, also plays a critical role in polymerization. GSH contains one cysteine and can easily be oxidized to form GSSG or protein bound glutathione (PSSG, Equation (1)) [112].

GSH + PSSP → PSSG + PSH(1)

That is to say, GSH may cleave the SS bonds of glutenin polymers and promote free cysteine formation, resulting in the trigger of SH/SS interchange reactions. Meanwhile, GSSG reacts with free sulfhydryl groups of aggregated glutenins during polymerization and generates PSSG. Finally, the average molecular weight of gluten network proteins decreases and dough softening occurs [112,113].

### 3.3. Secondary Structures of HMW-GS

The N- and C-terminal domains are both considered to be rich in α-helixes [114,115]. As for the central repetitive domains, β-reverse turns were initially proposed to be the dominant structural feature, as supported by the circular dichroism (CD) and Fourier transform infrared spectra (FTIR) study of synthetic peptides designed with the consensus peptides PGQGQQ and GYYPTSPQQ of HMW-GS [80,115,116,117]. These regularly repeated β-turns in the HMW-GS were organized into a β-spiral structure, supported by scanning tunneling microscopy images of 1Dx5 [116,118,119,120]. Analyses of the data from viscometric and small angle X-ray scattering also indicated that the structure of both reduced (no formation of disulfide bond) and alkylated HMW-GS are rod-shaped [119,120]. Despite β-turns, FTIR of HMW-GS in the hydrated solid state also indicated the presence of intermolecular β-sheet structures owing to the formation of extensive hydrogen bonds, which may be promoted by the N- and C-terminal domains [114,119,120]. The content of β-sheets was reported to change according to water content and temperature [121,122].

Although interchain disulfide bonds are crucial for stabilizing the HMW-GS polymers, nuclear magnetic resonance (NMR) studies indicate that hydrogen bonds mediated by glutamine side chains may also play an important role in stabilizing the gluten structure [121]. A “loop and train” model has been proposed for the contribution of such hydrogen bonding to gluten elasticity, in which HMW-GS has unbonded mobile regions (loops) and bonded regions (trains) at intermediate moisture contents [123]. The loops can be stretched, and when the stress is removed, reestablishment of the loop-train equilibrium provides the elastic restoring force; as a result, extension of the dough will result in stretching of the “loops” and “unzipping” of the “trains” [10]. The formation of interchain hydrogen bonds between glutamine residues may account for this observation as well [124,125]. Later, this model was further supported by many studies. NMR experiments showed that some parts of a purified HMW-GS chain were held in a much less mobile state, even at higher water contents [121]. According to the carbon and proton solid-state NMR study, regions rich in glutamine and glycine residues may form more mobile (or loop) parts of the network whereas regions containing high amount of hydrophobic residues intermingled with glutamines residues may form the junction zones (trains) [126]. In addition, the ratio of these “loops” and “trains” vary with the hydration level and with the length as well as sequence of the repetitive domain [121,127,128]. As the hydration level increases, the system is plasticized, allowing the orientation of β-turns in adjacent β-spirals to form structures that resemble an “interchain” β-sheet. Further extension will disrupt these “trains”, while release from the extension will result in reformation of the equilibrium balance, resulting in elastic recoil. Support for this hypothesis comes from a study in which changes in gluten-protein conformation were determined by FTIR spectroscopy during protein deformation [128].

## 4. Relationship to End Use Qualities

It has been reported that different HMW-GSs affect the proportion of gluten secondary structures, and higher contents of β-sheets and β-turns of gluten are generally considered to be associated with better dough qualities [101,108,122,129]. A near-isogenic line with the highest content of β-turns was reported to yield the greatest dough viscoelasticity, while another with the highest content of β-sheets exhibited the greatest wheat dough strength. Meanwhile, it was also mentioned that significant differences in the secondary structures of different wheat lines were mainly caused by their HMW-GSs [129]. Similar results from another research reported that the proportion of secondary structure of gluten in three wheat near-isogenic lines mainly resulted from the different HMW-GS compositions encoded by *Glu-A1* and *Glu-D1* loci and that the content of β-sheets in gluten has a significant relationship with dough rheological properties [101]. Compared with the normal Bx7 subunits, Bx7^OE^ subunits of a wheat near-isogenic line were also reported to yield increased content of β-sheets in gluten secondary structure and were related to superior dough rheological properties [130]. Besides, HMW-GSs with higher content of β-strands, indicated by secondary structure prediction, may be helpful to form a better gluten structure [131]. A longer repetitive domain or a higher proportion of repeats units of HMW-GSs may produce more β-turns, which confer elasticity to the polymers and are supposed to be positively related to dough and bread-making qualities [33,132,133,134,135,136].

Currently, scholars hold different views on the effect of α-helixes content on the dough quality. Compared with subunits from hexaploid wheat, subunits from TD-256, a cultivar with poor flour quality, possess less α-helixes quantity according to a secondary structure prediction study [111]. This indicated that the α-helixes content of HMW-GSs may exhibit positive effects on dough quality, supported by many other studies [131,133]. However, the content of α-helixes of gluten was reported to be negatively correlated with that of β-sheets, which could be explained by the transformation between α-helixes and β-sheets under certain conditions [122,129]. More α-helixes lead to less β-sheets, and the latter correlates positively with dough quality. Scholars further argue that α-helix content showed a negative correlation with dough quality [122]. In fact, significant correlations were found between the percentage of unextractable polymeric protein and the secondary structures mentioned above, so these secondary structures could be used as indicators for end product quality of dough [137].

## 5. Conclusions

HMW-GSs of wheat grain play a key role in determining rheological dough properties and end use properties. Both the interchain disulfide bonds and hydrogen bonds mediated by glutamine side chains are crucial for stabilizing the gluten structure. Specific alleles encoding HMW-GSs make greater contributions. In most cases, HMW-GSs with additional or less cysteines were supposed to give rise to relatively more or less formation of interchain disulfide bonds. As for secondary structures, HMW-GSs affect dough qualities by acting on the secondary structures’ composition of gluten. Though cumulative work has been carried out for the relationship between the genetics/structures of HMW-GSs and dough end use qualities, it is essential to explore the mechanism of crosslinking via disulfide bonds among different types of HMW-GS and other gluten components more specifically to establish an accurate model for the role that HMW-GSs play in the dough. The number of cysteine residues, the length of the repetitive domain, and the secondary structure content could be used as predictors to evaluate the quality of novel HMW-GSs for molecular breeding. Meanwhile, more efforts should be made to transfer desirable *Glu-1* alleles to cultivated wheat via hybridization and chromosomal engineering approaches.

## Abbreviations

HMW-GS	High-molecular-weight glutenin subunit
SDS-PAGE	Sodium dodecyl sulfate-polyacrylamide gel electrophoresis
LMW-GS	Low-molecular-weight glutenin subunit
PCR	Polymerase chain reaction
GUS	β-glucuronidase
Wis	Wheat insertion sequence
LTR	Long terminal repeat
1Dx5-N	N-terminal domain of 1Dx5
LC-MS/MS	liquid chromatograph-mass spectrometer/mass spectrometry
GSH	glutathione
PSSG	protein bound glutathione
FTIR	Fourier transform infrared spectroscopy
NMR	Nuclear magnetic resonance
